# Patterns of early life body mass index and childhood overweight and obesity status at eight years of age

**DOI:** 10.1186/s12887-018-1124-9

**Published:** 2018-05-11

**Authors:** Joseph M. Braun, Heidi J. Kalkwarf, George D. Papandonatos, Aimin Chen, Bruce P. Lanphear

**Affiliations:** 1 Department of Epidemiology, Brown University, Box G-S121-2, Providence, RI 02912 USA; 2 Department of Pediatrics, Cincinnati Children’s Hospital Medical Center, Cincinnati, OH 45229 USA; 3 Department of Biostatistics, Brown University, Providence, RI 02912 USA; 4 Department of Environmental Health, University of Cincinnati, Cincinnati, OH 45267 USA; 5 Child and Family Research Institute, BC Children’s and Women’s Hospital, Vancouver, BC Canada

**Keywords:** Adiposity, Children, Epidemiology, Obesity, And rapid growth

## Abstract

**Background:**

Excess weight gain in infancy and childhood is associated with increased risk of subsequent obesity. Identifying patterns of infancy and childhood weight gain associated with subsequent obesity or overweight status could help identify children at highest risk. Thus, we examined patterns of infancy and early childhood BMI in relation to mid-childhood overweight and obesity status.

**Methods:**

In a prospective cohort of 215 children from Cincinnati, OH (born: 2003–2006), we measured weight and length or height at ages 4 weeks and 1, 2, 3, 4, 5, and 8 years. We calculated BMI z-scores using World Health Organization references. Using linear fixed effect models, we estimated mean BMI at each age and rates of change in BMI between ages 4 weeks and 5 years by children’s overweight and obesity status at age 8 years, assessed with BMI z-scores or bioelectric impedance analysis (BIA).

**Results:**

Children who became overweight (BMI, *n* = 51 and BIA, *n* = 37) or obese (BMI, *n* = 22 and BIA, *n* = 29) at age 8 years had greater BMI at all ages compared to normal weightchildren. Children who were overweight had similar rates of change in BMI as children who were lean. Children who were obese had greater gains in BMI between age 4 weeks and 5 years, with the most rapid gains in the first 2 years.

**Conclusions:**

Results from this study of modest sample size, suggest that adiposity patterns in the first 5 years of life are related to subsequent childhood overweight and obesity risk.

**Electronic supplementary material:**

The online version of this article (10.1186/s12887-018-1124-9) contains supplementary material, which is available to authorized users.

## Background

Childhood obesity is a major global public health threat. Globally, 5.6% of girls and 7.8% of boys were obese in 2016 [1]. In the United States (US), 17% of children were obese and another 15% were overweight in 2010 [2]. Childhood obesity increases the risk of type 2 diabetes, cardiovascular disease, metabolic syndrome, and later life obesity, and has adverse effects on pulmonary, musculoskeletal, and psychosocial functioning [3–5]. There is a compelling need to prevent childhood obesity, since there are few effective non-pharmacological or non-surgical interventions to reduce excess adiposity [6].

Understanding the natural course of adiposity during infancy and childhood may help identify obesity risk factors or periods of heightened susceptibility. Previous studies have reported that earlier adiposity rebound, crossing major weight centiles during the first 2 years of life, and trajectories of rapid weight gain during infancy and childhood BMI are associated with increased childhood and adulthood adiposity and risk of obesity [5, 7–17]. However, few studies have examined these patterns among US children using measures of body composition, like bioelectric impedance, to define later childhood overweight or obesity status [13].

To improve our understanding of the patterns of adiposity associated with risk of childhood obesity and overweight among US children, we used data from 215 children with 1070 serial anthropometry measures from ages 4 weeks to 5 years to determine if overweight or obese status at age 8 years was associated with BMI or changes in BMI in the first 5 years of life. We hypothesized that children who were overweight or obese at age 8 years would have more rapid increases in BMI in the first 5 years of life compared to children who were lean.

## Methods

### Study participants

We used data from the Health Outcomes and Measures of the Environment (HOME) Study, a prospective cohort study designed to examine if and how early life environmental chemical exposures influence childhood health. Details regarding eligibility criteria, enrollment, follow-up, and assessments have been previously published [18]. Between March 2003 and January 2006, we recruited pregnant women from nine prenatal care clinics affiliated with three Cincinnati, Ohio area hospitals. At enrollment, women were eligible for the study if they were: 1) ≥ 18 years old, 2) living in a home built before 1978, 3) 16 ± 3 weeks of gestation, 4) no history of HIV infection, and 5) not taking any medications to treat thyroid disorders and seizure. All women provided written informed consent for themselves and their children after the study protocols were explained by research assistants. This study was approved by the institutional review boards of the Cincinnati Children’s Hospital Medical Center and cooperating delivery hospitals.

Of 389 live-born singletons, 219 (56%) completed the follow-up visit at age 8 years and had anthropometry data from this visit and at least one visit before age 8 years (Additional file 1: Supplemental Figure 1). Four children were missing relevant covariate data, leaving 215 children who provided 1070 repeated anthropometry measures for this analysis.

As we previously reported, participants who completed follow-up at age 8 years were similar to the original cohort in terms of baseline characteristics [18]. Most baseline characteristics of mother-child pairs who did and did not complete follow-up at age 8 years were similar, although there was a greater proportion of non-Hispanic Black women who completed follow-up than those who did not complete follow-up (36 vs. 27%) (Additional file 1: Supplemental Table 1).

### Anthropometry measurements

Trained research assistants obtained anthropometric measurements when children were 4 weeks and 1, 2, 3, 4, 5, and 8 years old. At all seven visits, we measured weight to the nearest 0.01 kg with the child dressed in a dry diaper or undergarments using a ScaleTronix scale. At 4 weeks and 1 year of age we measured length to `the nearest 0.1 cm using a length board. At or after age 2 years we measured height to the nearest 0.1 cm using an Ayrton Stadiometer (Model S100) with the child standing straight with heels positioned against the wall without shoes or head coverings. Fifty-five percent of children had all 6 BMI measurements, with 92% having ≥ 3 measures. At age 8 years, we used a Tanita children’s body fat monitor (BF-689) to estimate children’s body fat via bioelectric impedance analysis (BIA).

We calculated age- and sex-specific BMI z-scores at all ages using World Health Organization (WHO) references [19, 20]. At age 8 years, we classified children as normal weight, overweight, or obese using WHO references or bioelectric impedance analysis (BIA) [19, 21]. For BMI, we defined normal weight (< 1 standard deviation score), overweight (> 1 to ≤ 2 standard deviation scores), or obese (> 2 standard deviation scores) status at age 8 years using WHO definitions [19]. For BIA, we defined normal weight, overweight, and obese according to age- and sex-specific reference values [21]. We used both BMI and BIA at age 8 years to assess overweight and obesity status because BMI may not accurately reflect adipose tissue mass during childhood [22].

### Statistical analyses

We began by describing maternal and child characteristics among children who were normal weight vs. overweight or obese at age 8 years. These characteristics included maternal race (non-Hispanic White, non-Hispanic Black, and Other), education (high school or less, some college, and college or greater), marital status (married and unmarried), household income (continuous, US dollars per year), child sex (male and female), and duration of breastfeeding (continuous months). These variables were collected using standardized interviews that were administered by trained research assistants.

Next, we used linear regression models estimated via generalized least squares to estimate mean BMI z-scores and longitudinal changes in BMI z-scores between age 4 weeks and 5 years according to whether children were normal weight, overweight, or obese according to their age 8 year BMI or BIA value. We used the Akaike Information Criterion to identify the best fitting model and modeled BMI z-scores as a function of age, age-squared, and age-cubed terms with an unstructured covariance matrix and no random effects. Specifically, our model took the form:

$$ {\mathrm{Y}}_{\mathrm{ij}}={\upbeta}_{\mathrm{o}}+{\upbeta}_1{\mathrm{Age}}_{\mathrm{ij}}+{\upbeta}_2{\mathrm{Age}}_{\mathrm{ij}}^2+{\upbeta}_3{\mathrm{Age}}_{\mathrm{ij}}^3+\mathrm{OW}\left({\upbeta}_4+{\upbeta}_5{\mathrm{Age}}_{\mathrm{ij}}+{\upbeta}_6{\mathrm{Age}}_{\mathrm{ij}}^2+{\upbeta}_7{\mathrm{Age}}_{\mathrm{ij}}^3\right)+\mathrm{OB}\left({\upbeta}_8+{\upbeta}_9{\mathrm{Age}}_{\mathrm{ij}}+{\upbeta}_{10}{\mathrm{Age}}_{\mathrm{ij}}^2+{\upbeta}_{11}{\mathrm{Age}}_{\mathrm{ij}}^3\right)+{\upbeta}_{\mathrm{k}}{\mathrm{X}}_{\mathrm{k}} $$

Where Y_ij_ is the BMI z-score for the i-th child at the j-th visit, Age_ij_ is the child age for the i-th child at the j-th visit, OB and OW were indicator terms for overweight and obesity status at age 8 years, and β_k_X_k_ are additional terms that control for the aforementioned maternal and child characteristics. With this model, we estimated the mean BMI z-score at each age, as well as the annual rate of change in BMI z-score between adjacent measurements in children who were normal weight, overweight, or obese at age 8 years. We used Wald χ^2^ tests with 4 degrees of freedom to test whether BMI z-score trajectories differed by obesity status at age 8 years. Setting β_4_ = β_5_ = β_6_ = β_7_= 0 allowed us to compare normal weight vs. overweight trajectories, setting β_8_ = β_9_ = β_10_ = β_11_= 0 to compare normal weight vs. obese trajectories, and setting β_4_ − β_8_ = β_5_ − β_9_ = β_6_ − β_10_ = β_7_ − β_11_=0 to compare overweight and obese trajectories. Because of possible departures from normality induced by selection on the distal outcome (adiposity at age 8 years), all test procedures used robust standard error estimation in 95% confidence interval (CI) and *p*-value calculations.

### Sensitivity analyses

We conducted several sensitivity analyses to verify the robustness of our results. First, we examined whether BMI z-score trajectories varied by child sex, because boys and girls may accrue fat mass at different rates. Second, we excluded infants who were born small for gestational age (*n* = 23, birth weight < 10th percentile for gestational age and sex) or preterm (*n* = 18, < 37 weeks gestation) [23].

## Results

At age 8 years, 51 (24%) children were overweight, but not obese, and 22 (10%) children were obese, according to WHO definitions. Compared to children who were normal weight at age 8 years, children who were overweight or obese were more likely to have been breastfed for 0 to < 6 months and have mothers who were younger at delivery, non-Hispanic Black, less educated, poorer, and unmarried (Table 1). From ages 4 weeks to 5 years, 8.2–23.2% of children had a BMI z-score > 1 and 1.4–9.4% of children had a BMI z-score > 2 according to WHO definitions (Additional file 1: Supplemental Table 2). A total of 37 (17%) children were overweight and 29 (13%) were obese at age 8 years according to BIA-derived body fat percent.

**Table 1:** Baseline characteristics of mother-child pairs in the HOME Study according to the child’s overweight/obesity status at 8 years of age^a^

Variable	Number Normal Weight (%)	Number Overweight or Obese (%)	χ^2^ *p*-value^b^
All	142	73	
Maternal Age at Delivery			0.28
18- < 25 years	34 (24)	22 (30)	
25- < 34 years	84 (59)	44 (60)	
35+ years	24 (17)	7 (10)	
Maternal Race			< 0.01
Non-Hispanic White	94 (66)	33 (45)	
Non-Hispanic Black	41 (29)	36 (49)	
Other	7 (5)	4 (6)	
Household Income			0.11
<$20,000/year	32 (23)	27 (37)	
$20,000- < 40,000/year	21 (15)	12 (16)	
$40,000- < 80,000/year	49 (35)	20 (27)	
≥$80,000/year	40 (28)	14 (19)	
Maternal Education			0.01
<=High School	28 (20)	27 (37)	
Some College	47 (33)	15 (21)	
≥ College	67 (47)	31 (42)	
Maternal Marital Status			0.02
Unmarried	47 (33)	36 (49)	
Married	95 (67)	37 (51)	
Child Sex			0.45
Female	76 (54)	43 (59)	
Male	66 (46)	30 (41)	
Breastfeeding Duration			0.11
< 6 months	75 (53)	47 (64)	
≥ 6 months	67 (47)	26 (36)	

^a^Overweight or obesity were defined as having age- and sex-standardized BMI z-scores > 1 according to WHO reference data

^b^ χ^2^ *p*-value for the proportion of children who were overweight/obese vs. normal weight across covariate categories (k-1 degree of freedom test where k is the number of covariate categories)

**Fig. 1:**
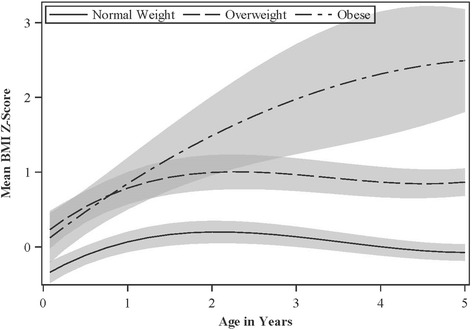
Adjusted Mean BMI Z-scores from Ages 4 Weeks to 5 Years According to WHO BMI-Derived Child Overweight/Obesity Status at Age 8 Years: The HOME Study ^a,b,c,d^ ^a^Adjusted for maternal race, education, marital status, age at delivery, household income, and breastfeeding duration ^b^Overweight and obesity status at age 8 years was defined as having age- and sex-specific BMI z-scores >1 and >2 according to WHO references, respectively ^c^142, 51, and 22 children were lean, overweight, or obese at age 8 years, respectively, and they contributed 732, 235, and 103 repeated BMI z-scores ^d^Shading represents the 95% confidence interval

**Fig. 2:**
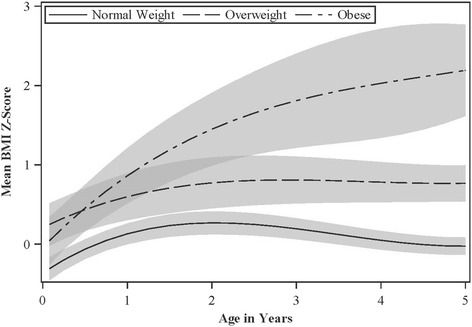
Adjusted Mean BMI Z-scores from Ages 4 Weeks to 5 Years According to Bioelectric Impedance-Derived Child Overweight/Obesity Status at Age 8 Years: The HOME Study ^a,b,c,d^ ^a^Adjusted for maternal race, education, marital status, age at delivery, household income, and breastfeeding duration ^b^Overweight and obesity status at age 8 years was defined as having age- and sex-specific body fat percent standard deviation scores >1 and >2, respectively [21] ^c^149, 37, and 29 children were lean, overweight, or obese at age 8 years, respectively, and they contributed 761, 177, and 132 repeated BMI z-scores ^d^Shading represents the 95% confidence interval

The pattern of BMI z-scores among children who became obese at age 8 years was significantly different compared to children who were normal weight or overweight at age 8 years (Wald χ^2^ *p*-values< 0.001). Compared to children who were normal weight at age 8 years, children who were obese had higher BMI z-scores at all ages, including age 4-weeks, and the magnitude of these differences increased as children aged (Tables 2 and 3, Figs. 1 and 2, and Additional file 1: Supplemental Tables 3 and 4). Children who became obese at age 8 years had positive rates of BMI z-score change at all ages, but the annual rates of change were greater between 4 weeks and 2 years than between 2 and 5 years.

**Table 2:** Model-derived adjusted mean and difference in BMI z-score and annual rate of BMI z-score change from 4-weeks to 5 years of age according to children’s overweight/obesity status at 8 years of age: The HOME Study ^a, b, c^

Group-Time	Mean BMI Z-Score (95% CI)	Difference in BMI Z-score vs. Normal Weight (95% CI)^d^	*p*-value^e^	Difference in BMI Z-score vs. Overweight (95% CI)^f^	*p*-value^g^	Annual BMI Z-score Change (95% CI)^h^	*p*-value^i^	*p*-value^j^
Normal Weight-4 weeks	− 0.34 (− 0.48, − 0.20)	Ref	N/A	N/A	N/A	N/A	N/A	N/A
Normal Weight-1 year	0.07 (− 0.07, 0.20)	Ref	N/A	N/A	N/A	0.41 (0.28, 0.53)	N/A	N/A
Normal Weight-2 year	0.20 (0.05, 0.35)	Ref	N/A	N/A	N/A	0.13 (0.09, 0.18)	N/A	N/A
Normal Weight-3 year	0.14 (0, 0.27)	Ref	N/A	N/A	N/A	−0.06 (−0.11, −0.02)	N/A	N/A
Normal Weight-4 year	0 (−0.12, 0.12)	Ref	N/A	N/A	N/A	−0.13 (− 0.19, − 0.08)	N/A	N/A
Normal Weight-5 year	− 0.08 (− 0.19, 0.03)	Ref	N/A	N/A	N/A	−0.08 (− 0.13, − 0.02)	N/A	N/A
Overweight-4 weeks	0.23 (− 0.02, 0.48)	0.57 (0.29, 0.86)	0.0001	Ref	N/A	N/A	N/A	N/A
Overweight-1 year	0.79 (0.57, 1.00)	0.72 (0.47, 0.97)	<.0001	Ref	N/A	0.55 (0.34, 0.76)	0.2403	N/A
Overweight-2 year	1.00 (0.76, 1.23)	0.8 (0.53, 1.07)	<.0001	Ref	N/A	0.21 (0.13, 0.30)	0.115	N/A
Overweight-3 year	0.97 (0.75, 1.18)	0.83 (0.58, 1.08)	<.0001	Ref	N/A	−0.03 (−0.11, 0.05)	0.4678	N/A
Overweight-4 year	0.87 (0.66, 1.07)	0.87 (0.64, 1.09)	<.0001	Ref	N/A	−0.1 (− 0.18, − 0.02)	0.4863	N/A
Overweight-5 year	0.87 (0.68, 1.05)	0.94 (0.73, 1.15)	<.0001	Ref	N/A	0 (−0.09, 0.09)	0.1706	N/A
Obese-4 weeks	0.12 (−0.21, 0.45)	0.46 (0.10, 0.82)	0.0131	−0.11 (− 0.53, 0.31)	0.6072	N/A	N/A	N/A
Obese-1 year	0.85 (0.50, 1.19)	0.78 (0.41, 1.15)	<.0001	0.06 (−0.35, 0.47)	0.7728	0.73 (0.44, 1.01)	0.0477	0.3459
Obese-2 year	1.49 (0.96, 2.02)	1.29 (0.74, 1.84)	<.0001	0.49 (−0.09, 1.07)	0.0975	0.64 (0.37, 0.91)	0.0003	0.0032
Obese-3 year	1.98 (1.24, 2.71)	1.84 (1.10, 2.59)	<.0001	1.01 (0.24, 1.78)	0.0105	0.49 (0.21, 0.77)	0.0002	0.0005
Obese-4 year	2.31 (1.48, 3.15)	2.31 (1.47, 3.16)	<.0001	1.45 (0.59, 2.31)	0.0012	0.34 (0.16, 0.51)	<.0001	<.0001
Obese-5 year	2.49 (1.81, 3.18)	2.57 (1.87, 3.27)	<.0001	1.63 (0.92, 2.34)	< 0.0001	0.18 (−0.05, 0.41)	0.0344	0.1593

^a^Adjusted for maternal race, education, marital status, age at delivery, household income, and breastfeeding duration

^b^Overweight and obesity status at age 8 years was defined as having age- and sex-specific BMI z-scores > 1 and > 2 according to WHO references, respectively

^c^142, 51, and 22 children were normal weight, overweight, or obese, respectively, and they contributed 732, 235, and 103 repeated BMI z-scores

^d^Adjusted mean difference in BMI z-score at each age among children who were obese or overweight at age 8 years vs. children who were normal weight at age 8 years

^e^ *p*-value for the mean difference in BMI z-score at each age among children who were obese or overweight at age 8 years vs. children who were normal weight at age 8 years

^f^Adjusted mean difference in BMI z-score at each age among children who were obese at age 8 years vs. children who were overweight at age 8 years

^g^*p*-value for the mean difference in BMI z-score at each age among children who were obese at age 8 years vs. children who were overweight at age 8 years

^h^Adjusted annual rate of change in BMI z-score between the prior measurement and the current measurement

^i^*p*-value for the difference in the adjusted annual rate of change in BMI z-score among children who were obese or overweight at age 8 years vs. children who were normal weight at age 8 years

^j^*p*-value for the difference in the adjusted annual rate of change in BMI z-score among children who were obese at age 8 years vs. children who were overweight at age 8 years

**Table 3:** Model-derived adjusted mean and difference in BMI z-score and annual rate of BMI z-score change from 4-weeks to 5 years of age according to children’s overweight/obesity status at 8 years of age measured by bioelectic impedance: The HOME Study ^a, b, c^

Group-Time	Mean BMI Z-Score (95% CI)	Difference in BMI Z-score vs. Normal Weight (95% CI)^d^	*p*-value^e^	Difference in BMI Z-score vs. Overweight (95% CI)^f^	*p*-value^g^	Annual BMI Z-score Change (95% CI)^h^	*p*-value^i^	*p*-value^j^
Normal Weight-4 weeks	− 0.31 (− 0.46, − 0.17)	Ref	N/A	N/A	N/A	N/A	N/A	N/A
Normal Weight-1 year	0.13 (0, 0.26)	Ref	N/A	N/A	N/A	0.44 (0.31, 0.56)	N/A	N/A
Normal Weight-2 year	0.27 (0.12, 0.41)	Ref	N/A	N/A	N/A	0.14 (0.09, 0.19)	N/A	N/A
Normal Weight-3 year	0.19 (0.06, 0.32)	Ref	N/A	N/A	N/A	−0.07 (− 0.12, − 0.03)	N/A	N/A
Normal Weight-4 year	0.05 (− 0.07, 0.16)	Ref	N/A	N/A	N/A	−0.15 (− 0.19, − 0.1)	N/A	N/A
Normal Weight-5 year	− 0.03 (− 0.14, 0.08)	Ref	N/A	N/A	N/A	−0.07 (− 0.13, − 0.02)	N/A	N/A
Overweight-4 weeks	0.25 (− 0.02, 0.52)	0.56 (0.25, 0.86)	0.0004	Ref	N/A	N/A	N/A	N/A
Overweight-1 year	0.6 (0.3, 0.89)	0.47 (0.15, 0.79)	0.0047	Ref	N/A	0.35 (0.15, 0.55)	0.4593	N/A
Overweight-2 year	0.77 (0.45, 1.09)	0.51 (0.15, 0.86)	0.0052	Ref	N/A	0.17 (0.1, 0.25)	0.4309	N/A
Overweight-3 year	0.81 (0.51, 1.1)	0.62 (0.29, 0.94)	0.0002	Ref	N/A	0.04 (−0.04, 0.11)	0.01	N/A
Overweight-4 year	0.78 (0.53, 1.03)	0.73 (0.46, 1.01)	< 0.0001	Ref	N/A	−0.03 (− 0.12, 0.06)	0.0242	N/A
Overweight-5 year	0.77 (0.54, 0.99)	0.79 (0.54, 1.04)	< 0.0001	Ref	N/A	−0.01 (− 0.13, 0.11)	0.3777	N/A
Obese-4 weeks	0.04 (−0.26, 0.34)	0.35 (0.01, 0.69)	0.042	−0.21 (− 0.61, 0.2)	0.3193	N/A	N/A	N/A
Obese-1 year	0.86 (0.51, 1.21)	0.73 (0.36, 1.1)	0.0001	0.26 (−0.19, 0.72)	0.2602	0.82 (0.52, 1.12)	0.0235	0.0116
Obese-2 year	1.45 (0.99, 1.91)	1.18 (0.7, 1.66)	< 0.0001	0.68 (0.12, 1.24)	0.0187	0.59 (0.37, 0.81)	0.0001	0.0006
Obese-3 year	1.81 (1.21, 2.4)	1.62 (1.01, 2.22)	< 0.0001	1.00 (0.34, 1.66)	0.0034	0.36 (0.09, 0.63)	0.0021	0.0242
Obese-4 year	2.03 (1.34, 2.72)	1.98 (1.28, 2.68)	< 0.0001	1.25 (0.52, 1.98)	0.001	0.22 (0.02, 0.42)	0.0007	0.0308
Obese-5 year	2.19 (1.61, 2.77)	2.22 (1.63, 2.81)	< 0.0001	1.42 (0.81, 2.04)	< 0.0001	0.16 (−0.04, 0.36)	0.0258	0.1387

^a^Adjusted for maternal race, education, marital status, age at delivery, household income, and breastfeeding duration

^b^Overweight and obesity status at age 8 years was defined as having age- and sex-specific body fat percent standard deviation scores > 1 and > 2, respectively [23].

^c^149, 37, and 29 children were normal weight, overweight, or obese at age 8 years, respectively, and they contributed 761, 177, and 132 repeated BMI z-scores

^d^Adjusted mean difference in BMI z-score at each age among children who were obese or overweight at age 8 years vs. children who were normal weight at age 8 years

^e^*p*-value for the mean difference in BMI z-score at each age among children who were obese or overweight at age 8 years vs. children who were normal weight at age 8 years

^f^ Adjusted mean difference in BMI z-score at each age among children who were obese at age 8 years vs. children who were overweight at age 8 years

^g^*p*-value for the mean difference in BMI z-score at each age among children who were obese at age 8 years vs. children who were overweight at age 8 years

^h^Adjusted annual rate of change in BMI z-score between the prior measurement and the current measurement

^i^*p*-value for the difference in the adjusted annual rate of change in BMI z-score among children who were obese or overweight at age 8 years vs. children who were normal weight at age 8 years

^j^*p*-value for the difference in the adjusted annual rate of change in BMI z-score among children who were obese at age 8 years vs. children who were overweight at age 8 years

Children who were overweight at age 8 years had statistically significantly higher BMI z-scores than normal weight children at all ages, but their pattern of change in BMI z-scores was not significantly different than normal weight children (Wald χ^2^ *p*-value = 0.84). Children who became overweight at age 8 years had similar BMI z-scores as obese children at ages 4 weeks and 1 and 2 years, but their mean BMI z-score plateaued after age 2 years and was statistically significantly lower at ages 3, 4, and 5 years compared to children who became obese (Tables 2 and 3). The annual rates of change in BMI z-scores among children who became overweight at age 8 years were positive before age 2 years and then approximately 0 after age 2 years (Tables 2 and 3).

When we examined BMI z-score trajectories according to normal weight, overweight, and obesity status at age 8 years assessed with BIA, our results were similar to those when we used BMI z-score to define adiposity at age 8 years (Fig. 2, Table 3, Additional file 1: Supplemental Table 4). There were no notable differences in the patterns of child BMI z-score by overweight/obesity status when we stratified by child sex (results not shown). Our results were not appreciably different when we excluded children who were small for gestational age or preterm (results not shown).

## Discussion

In this cohort, we observed that children who were overweight or obese at age 8 years had higher BMI z-scores between 4 weeks and 5 years of age than their normal weight peers. Compared to children who were normal weight or overweight at age 8 years, children who were obese had greater gains in BMI during the first 5 years of life with the greatest gains in BMI during the first 2 years of life. Children who were overweight had similar rates of BMI gains as children who were normal weight, but they had higher BMI than normal weight children at all ages, including at age 4 weeks.

Our finding of more rapid gains in adiposity in the first 2 years of life among children who became obese is consistent with prior studies observing increased risk of later life obesity among infants who grow rapidly in the first 2 years of life [8–11, 24]. Additional studies have tried to identify patterns and trajectories of early childhood adiposity associated with risk of obesity in child- or adulthood with relatively consistent results [12–15, 25]. Our results are generally consistent with the results of most prior studies [12, 13, 15, 25]. Glavin et al. found an increased risk of obesity and overweight at age 8 years among Norwegian children with more rapid gains in adiposity during infancy and more adiposity in later childhood, findings consistent with our own [12]. In a study of US children, greater gains in adiposity in the first 3 years of life were associated with measures of body composition at age 6–10 years; these associations were strongest for adiposity gains between age 2–3 years [13]. A study of French adults reported that BMI trajectories between birth and age 10 years characterized by above average adiposity were positively associated with both BMI and waist circumference in adulthood [15]. In a longitudinal study of Chilean children, prevalent, but not incident obesity and overweight status in the first 4 years of life was associated with increased risk of being overweight or obese at age 7 years [25]. Finally, and in contrast to the current and prior work, BMI in adults born and living in India was more strongly correlated with BMI and increases in BMI in later childhood [14]. However, a large proportion of this cohort was underweight during childhood, which may explain these results.

Children who became overweight in this cohort did not experience more rapid gains in BMI in the first 5 years of life compared to lean children; however, they did have higher BMI at all ages, with these elevations becoming relatively stable at ages 3, 4, and 5 years. A prior study reported that children who were overweight at age 5 years had 4-times the risk of being obese by age 14 years compared to children who were lean [5]. Thus, children who are overweight in early childhood, but not obese, represent a susceptible sub-population who are at higher risk of developing obesity. Additional research is necessary to understand factors that increase the risk of this transition [26].

The causal link between rapid gains in adiposity during infancy and risk of obesity is not clear. Cole has argued that this is a statistical phenomenon and not a physiological one, as individuals who are obese later in life are more likely to have a higher weight earlier in life and more likely to be crossing major weight centiles earlier in life [27]. Even if rapid gains in adiposity during infancy are not causally related to obesity risk, they may be useful clinical markers for predicting subsequent risk of obesity [10]; however, additional work is needed to confirm this as simple measures of childhood adiposity may not have strong predictive value for adult adiposity or disease risk [28].

Our study has several strengths and limitations. First, we used prospectively collected and longitudinal research-quality anthropometry measurements to characterize children’s BMI trajectories. In addition, we used BMI and BIA to define overweight and obesity status at age 8 years. However, our sample size was modest when examining the number of children who were obese or overweight at age 8 years. Despite this, we observed large and statistically significant, albeit imprecise, differences in mean BMI z-scores or rates of BMI z-score change when comparing children who were obese, overweight, or normal weightat age 8 years. Second, we used linear fixed effect models to characterize average BMI and rates of BMI change over the first 5 years of life according to age 8-year adiposity status. While prior studies have classified longitudinal measures of adiposity and related these to subsequent adiposity status, it was unlikely that our sample size was large enough to identify unique classes with sufficient sample size [29]. Indeed, prior studies using these methods with larger sample sizes (*n* > 1000) still observed relatively small cell sizes for some trajectories of adiposity (e.g., *n* < 20) [15]. Third, while we adjusted for several determinants of childhood obesity, we could not adjust for child diet or physical activity. In addition, our modest sample size precluded us from examining differences in subgroups of children; however, we did observe similar patterns of adiposity gain among children without in utero growth restriction or who were born at term. Fourth, we examined a group of healthy and typically developing US children. Still, our findings may not be generalizable to other populations if there are environmental or genetic factors that modify the relations between growth and risk of childhood obesity. Finally, we examined several features of early childhood growth and some of our results may be chance findings. However, these features were consistent when we used either BMI or BIA to define overweight/obesity status at age 8 years, thus reducing the possibility of spurious results.

## Conclusions

Our results, from this modestly sized cohort, contribute to the body of literature suggesting that the risk of childhood obesity has origins in early in life. Efforts to identify interventions to prevent childhood obesity should focus on the periods of gestation and infancy as obesity risk may be malleable during these periods.

## Additional file

Additional file 1: **Supplemental Table 1.** Baseline characteristics of mother-child pairs in the HOME Study according inclusion vs. exclusion status. **Supplemental Table 2.** Proportion of children who were overweight or obese at ages 4 weeks to 5 years according to World Health Organization definitions^a^. **Supplemental Table 3.** Univariate Statistics of BMI Z-scores at each Study Visit According to Children’s Overweight/Obesity Status Assessed by WHO BMI z-score at Age 8 Years^a^. **Supplemental Table 4.** Univariate Statistics of BMI Z-scores at each Study Visit According to Children’s Overweight/Obesity Status Assessed by Bioelectric Impedance at Age 8 Years^a^. **Supplemental Figure 1.** Flowchart describing study participant selection in present study. (DOCX 79 kb)

## Abbreviations

BMI: Body Mass Index
HOME: Health Outcomes and Measures of the Environment
IOTF: International Obesity Task Force
WHO: World Health Organization
