# Supplementary material for: Patterns of early life body mass index and childhood overweight and obesity status at eight years of age
Source: BMC Pediatr. 2018 May 11;18:161. doi: 10.1186/s12887-018-1124-9 (PMC5948906; doi:10.1186/s12887-018-1124-9)

**Supplemental Material For:** Patterns of Early Life Body Mass Index and Childhood Overweight and Obesity Status at Eight Years of Age

**Authors:** Joseph M. Braun,^1^ Heidi J. Kalkwarf,^2^ George D. Papandonatos,^3^ Aimin Chen,^4^ and Bruce Lanphear^5^

Contents

[Supplemental Table 1: Baseline characteristics of mother-child pairs in the HOME Study according inclusion vs. exclusion status. 2](#_Toc508375135)

[Supplemental Table 2: Proportion of children who were overweight or obese at ages 4 weeks to 5 years according to World Health Organization definitionsa 3](#_Toc508375136)

[Supplemental Table 3: Univariate Statistics of BMI Z-scores at each Study Visit According to Children’s Overweight/Obesity Status Assessed by WHO BMI z-score at Age 8 Years^a^ 4](#_Toc508375137)

[Supplemental Table 4: Univariate Statistics of BMI Z-scores at each Study Visit According to Children’s Overweight/Obesity Status Assessed by Bioelectric Impedance at Age 8 Years^a^ 5](#_Toc508375138)

[Supplemental Figure 1: Flowchart describing study participant selection in present study 6](#_Toc508375139)

# Supplemental Table 1: Baseline characteristics of mother-child pairs in the HOME Study according inclusion vs. exclusion status.

| **Variable** | **Excluded** | **Included** |
| --- | --- | --- |
| **All** | 174 | 215 |
| **Maternal Age at Delivery** |  |  |
| 18-<25 years | 38 (22) | 56 (26) |
| 25-<34 years | 101 (60) | 128 (60) |
| 35+ years | 30 (18) | 31 (14) |
| **Maternal Race** |  |  |
| Non-Hispanic White | 106 (65) | 127 (59) |
| Non-Hispanic Black | 44 (27) | 77 (36) |
| Other | 14 (8) | 11 (5) |
| **Household Income** |  |  |
| <$20,000/year | 29 (18) | 59 (27) |
| $20,000-<40,000/year | 32 (19) | 33 (15) |
| $40,000-<80,000/year | 57 (35) | 69 (32) |
| >$80,000/year | 46 (28) | 54 (25) |
| **Maternal Education** |  |  |
| <=High School | 40 (24) | 55 (26) |
| Some College | 35 (21) | 62 (29) |
| > College | 89 (54) | 98 (45) |
| **Maternal Marital Status** |  |  |
| Unmarried | 52 (32) | 83 (39) |
| Married | 112 (68) | 132 (61) |
| **Child Sex** |  |  |
| Female | 86 (51) | 119 (55) |
| Male | 83 (49) | 96 (45) |
| **Breastfeeding Duration** |  |  |
| <6 months | 111 (65) | 122 (57) |
| >6 months | 61 (35) | 93 (43) |

# Supplemental Table 2: Proportion of children who were overweight or obese at ages 4 weeks to 5 years according to World Health Organization definitions^a^

| Age | Overweight N (%) | Obese N (%) |
| --- | --- | --- |
| 4-Weeks | 17 (8.2) | 3 (1.4) |
| 1-Year | 46 (23.2) | 7 (3.5) |
| 2-Years | 42 (23.2) | 17 (9.4) |
| 3-Years | 31 (17.9) | 8 (4.6) |
| 4-Years | 22 (15.3) | 6 (4.2) |
| 5-Years | 28 (16.9) | 9 (5.4) |

a-Overweight defined as BMI z-score > 2 and <3. Obese defined as BMI z-score >3.

# Supplemental Table 3: Univariate Statistics of BMI Z-scores at each Study Visit According to Children’s Overweight/Obesity Status Assessed by WHO BMI z-score at Age 8 Years^a^

| **OW/OB Status at Age 8 Years and Age** |  | **N** | **Mean BMI Z-Score (SD)** | **Min, Max BMI Z-Score** |
| --- | --- | --- | --- | --- |
| Normal Weight-4 weeks |  | 139 | -0.35 (0.92) | -2.74, 2.46 |
| Normal Weight-1 year |  | 134 | 0.06 (0.96) | -2.80, 3.29 |
| Normal Weight-2 year |  | 120 | 0.20 (0.96) | -2.39, 2.78 |
| Normal Weight-3 year |  | 118 | 0 (0.88) | -2.17, 2.00 |
| Normal Weight-4 year |  | 104 | 0.05 (0.74) | -1.66, 2.06 |
| Normal Weight-5 year |  | 117 | -0.08 (0.73) | -1.77, 1.73 |
| Overweight-4 weeks |  | 48 | 0.20 (1.00) | -3.18, 2.21 |
| Overweight -1 year |  | 44 | 0.84 (0.77) | -0.75, 2.82 |
| Overweight -2 year |  | 41 | 1.10 (0.93) | -1.44, 3.14 |
| Overweight -3 year |  | 38 | 0.74 (0.96) | -2.7, 2.13 |
| Overweight -4 year |  | 30 | 0.90 (0.58) | -0.29, 2.12 |
| Overweight -5 year |  | 34 | 0.93 (0.59) | -0.08, 2.18 |
| Obese-4 weeks |  | 21 | 0.15 (0.80) | -1.64, 1.58 |
| Obese -1 year |  | 20 | 0.66 (0.97) | -1.12, 2.13 |
| Obese -2 year |  | 20 | 1.61 (1.25) | -0.10, 4.09 |
| Obese -3 year |  | 17 | 1.80 (1.74) | 0.15, 6.49 |
| Obese -4 year |  | 10 | 1.90 (1.90) | -0.16, 6.28 |
| Obese -5 year |  | 15 | 2.38 (1.14) | 0.41, 5.19 |

*-Children were on average (range) 1.1 (0.8-2.0), 13.1 (11.3-19.3), 25 (22-28.1), 37.2 (34.6-43.5), 49.5 (45.3-55.7), 62.1 (57.0-72.0), and 97.7 (90.1-120) months of age at the 4-week, 1-year, 2-year, 3-year, 4-year, 5-year, and 8-year study visits, respectively.

a-Overweight and obesity status at age 8 years was defined as having age- and sex-specific BMI z-scores >1 and >2 according to WHO references, respectively.

#

# Supplemental Table 4: Univariate Statistics of BMI Z-scores at each Study Visit According to Children’s Overweight/Obesity Status Assessed by Bioelectric Impedance at Age 8 Years^a^

| **OW/OB Status at Age 8 Years and Age** | **N** | **Mean BMI Z-Score (SD)** | **Min, Max BMI Z-Score** |
| --- | --- | --- | --- |
| Normal Weight-4 weeks | 145 | -0.29 (0.97) | -3.18, 2.18 |
| Normal Weight-1 year | 140 | 0.1 (0.93) | -2.8, 3.29 |
| Normal Weight-2 year | 128 | 0.32 (0.94) | -2.39, 2.78 |
| Normal Weight-3 year | 123 | 0.06 (0.88) | -2.17, 2.00 |
| Normal Weight-4 year | 105 | 0.07 (0.72) | -1.66, 2.06 |
| Normal Weight-5 year | 120 | -0.03 (0.74) | -1.77, 1.82 |
| Overweight-4 weeks | 36 | 0.13 (0.96) | -1.34, 2.46 |
| Overweight -1 year | 32 | 0.76 (0.88) | -1.38, 2.82 |
| Overweight -2 year | 28 | 0.69 (1.16) | -1.85, 3.14 |
| Overweight -3 year | 29 | 0.59 (1.05) | -2.7, 2.13 |
| Overweight -4 year | 25 | 0.77 (0.78) | -1.3, 2.12 |
| Overweight -5 year | 27 | 0.73 (0.76) | -1.15, 2.18 |
| Obese-4 weeks | 27 | 0.07 (0.79) | -1.64, 1.58 |
| Obese -1 year | 26 | 0.79 (1.01) | -1.12, 2.48 |
| Obese -2 year | 25 | 1.66 (1.24) | -0.81, 4.09 |
| Obese -3 year | 21 | 1.68 (1.65) | -0.12, 6.49 |
| Obese -4 year | 14 | 1.76 (1.58) | -0.16, 6.28 |
| Obese -5 year | 19 | 2.18 (1.09) | 0.41, 5.19 |

*-Children were on average (range) 1.1 (0.8-2.0), 13.1 (11.3-19.3), 25 (22-28.1), 37.2 (34.6-43.5), 49.5 (45.3-55.7), 62.1 (57.0-72.0), and 97.7 (90.1-120) months of age at the 4-week, 1-year, 2-year, 3-year, 4-year, 5-year, and 8-year study visits, respectively.

a- Overweight and obesity status at age 8 years was defined as having age- and sex-specific body fat percent standard deviation scores >1 and >2, respectively.

# Supplemental Figure 1: Flowchart describing study participant selection in present study


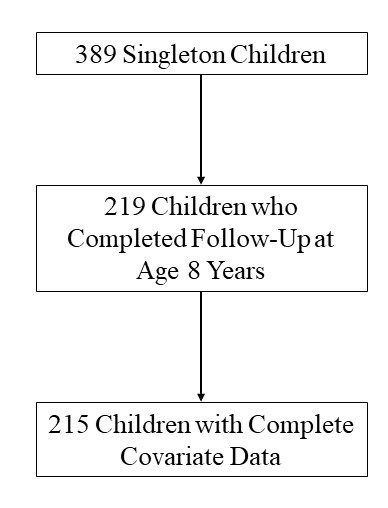

Supplement: Supplementary file 1 — Supplemental Table 1. Baseline characteristics of mother-child pairs in the HOME Study according inclusion vs. exclusion status. Supplemental Table 2. Proportion of children who were overweight or obese at ages 4 weeks to 5 years according to World Health Organization definitionsa. Supplemental Table 3. Univariate Statistics of BMI Z-scores at each Study Visit According to Children’s Overweight/Obesity Status Assessed by WHO BMI z-score at Age 8 Yearsa. Supplemental Table 4. Univariate Statistics of BMI Z-scores at each Study Visit According to Children’s Overweight/Obesity Status Assessed by Bioelectric Impedance at Age 8 Yearsa. Supplemental Figure 1. Flowchart describing study participant selection in present study. (DOCX 79 kb) [file 12887_2018_1124_MOESM1_ESM.docx]
